# A Scoping Review on Nutrition Knowledge and Nutrition Literacy Among Pregnant Women and the Prevalence of Pregnancy Complications and Adverse Pregnancy Outcomes

**DOI:** 10.3390/nu17213488

**Published:** 2025-11-06

**Authors:** Tinuola Oladebo, Faith Bobholz, Kevin Folivi, Julia Dickson-Gomez, Ronald Anguzu, Alexa A. Lopez, Idayat Akinola, Jessica Olson, Anna Palatnik

**Affiliations:** 1 Institute of Health and Humanity, Medical College of Wisconsin, Milwaukee, WI 53226, USAkfolivi@mcw.edu (K.F.); ranguzu@mcw.edu (R.A.);; 2 School of Nursing, University of Wisconsin-Milwaukee, Milwaukee, WI 53211, USA; 3 Department of Obstetrics and Gynecology, Division of Maternal Fetal Medicine, Medical College of Wisconsin, 9200 W Wisconsin Ave, Milwaukee, WI 53226, USA; 4 Cardiovascular Center, Medical College of Wisconsin, Milwaukee, WI 53226, USA

**Keywords:** pregnant women, adverse maternal outcomes, adverse neonatal outcomes, gestational diabetes mellitus, hypertensive disorders of pregnancy, nutrition, nutrition literacy, nutrition knowledge

## Abstract

**Background**: Proper nutrition supports maternal and fetal health. Gaps in nutritional knowledge (NK) and nutritional literacy (NL) can affect maternal and fetal health. NK refers to knowing facts and processes about nutrition, while NL is a broader component that includes competencies and skills needed to obtain, understand, and apply nutrition information to make dietary decisions. NL and NK limitations may contribute to adverse maternal and neonatal outcomes. This scoping review aims to understand the relationship between NK, NL, and pregnancy outcomes, offering insights into areas for future nutrition-based interventions. **Methods**: Seven databases were searched for studies assessing NK and NL among pregnant women. A total of 5080 articles were identified, with 4249 retained after removing duplicates. Following title and abstract screening, 18 articles underwent full-text review, and 11 met the inclusion criteria. Data were extracted, analyzed, and categorized into nine key themes. **Results**: All eleven studies employed survey-based methods; ten focused on NK and one on NL. Overall, NK was generally low. The evidence for an association between NK or NL and pregnancy outcomes was limited. Education, income, occupation, and family influence were identified as key factors influencing the NK and NL of pregnant women. Education and income levels were identified as having the most significant impact on NK overall. Only one study accessed the relationship between NK and adverse birth and neonatal outcomes, and this only included HDP and preterm labor. Also, only one out of the eleven studies was conducted in the US. **Conclusions**: In this review, we found that NK and NL among pregnant women was generally low, with limited evidence linking it to pregnancy outcomes; education and income emerged as the most influential factors of NK and NL. Future studies in high-income countries are recommended to assess the association between NL and adverse maternal outcomes, especially GDM.

## 1. Introduction

Pregnancy is a uniquely demanding physiological state characterized by profound anatomical, hormonal, and metabolic adaptations to support maternal and fetal health [1]. These dynamic changes, however, can predispose women to complications such as gestational diabetes mellitus (GDM) and hypertensive disorders of pregnancy (HDP). They may also influence fetal and neonatal development in ways that have lasting effects on health across the lifespan [1,2,3,4,5]. In the United States, the prevalence of GDM and HDP have increased substantially in recent years [6,7], paralleling the rise in modifiable risk factors such as obesity and sedentary lifestyle [6].

Despite the well-established link between nutrition and pregnancy health, many women face challenges in meeting dietary recommendations due to limited understanding or application of nutritional principles [8]. Nutritional knowledge (NK) refers to the factual understanding a pregnant woman has about nutrition—including awareness of dietary guidelines, nutrient sources, everyday food choices, and the relationship between diet and disease—and the ability to recognize, recall, and apply food- and nutrition-related terminology [8,9]. Nutritional literacy (NL), on the other hand, is the ability to interpret and use NK to make informed food choices and maintain a healthy diet. It encompasses practical skills and decision-making beyond basic knowledge [2,9,10]. Inadequate NK and NL may increase the risk of adverse maternal and neonatal outcomes [2,8]. One limitation in assessing NL is its frequent conflation with the broader concept of health literacy [11]. Health literacy is defined as the ability to obtain, process, and understand basic health information and services needed to make informed decisions about overall well-being and encompasses topics beyond nutrition, such as physical activity, disease prevention, and navigating healthcare systems [11].

Given these challenges, this scoping review aims to evaluate cross-sectional studies on NK and NL among pregnant women, identify the existing gaps, and guide future research and interventions designed to enhance pregnancy outcomes through improved nutritional understanding and application.

## 2. Materials

### 2.1. Theoretical Framework

This scoping review followed the six-stage methodological framework proposed by Arksey and O’Malley [12]. The process began with the identification of the research question (stage 1) and a comprehensive search for relevant studies (stage 2). Stage 3 involved selecting studies, followed by data charting in stage 4. In stage 5, the results were collated, summarized, and reported. Additionally, a study protocol was developed and registered [13] in accordance with Open Science Framework (OSF) guidelines for scoping reviews (https://osf.io/wkmpx, accessed on 25 September 2025). The review concluded in stage 6 with a team consensus on the research focus: evaluating studies assessing NK and NL among pregnant women.

### 2.2. Eligibility Criteria

The eligibility criteria for article inclusion for this study were as follows:

- Peer-reviewed articles;
- Written in English;
- Pregnant study participants;
- Studies assessing NK or NL in pregnant women;
- Exploratory studies;
- Studies using surveys with a list of items measuring NK or NL;
- Assessment of NK or NL, either as an outcome or an independent variable;
- Studies reported how NK or NL was scored.

### 2.3. Literature Search and Study Selection

A comprehensive search was conducted by a librarian across seven databases on 11 November 2024: PubMed, Scopus, Web of Science, ProQuest Dissertations & Theses via WOS, Health Source: Nursing/Academic Edition, Cochrane Reviews, and Cochrane Trials. The search utilized a variety of keywords, including “nutrition”, “nutrition knowledge”, nutrition literacy”, “prenatal nutritional phenomena”, “pregnancy complications”, “eclampsia”, “gestational diabetes”, and “adverse pregnancy outcomes”. The search terms used in the searches for each database are available in the Appendix A. The results from searches were organized and screened using Rayyan (new.rayyan.ai).

A total of 5080 results were retrieved across the seven databases: 1484 from PubMed, 413 from Scopus, 447 from Web of Science, 80 from ProQuest Dissertations & Theses via WOS, 137 from Health Source: Nursing/Academic Edition, 101 from Cochrane Reviews, and 2418 from Cochrane Trials. After removing 831 duplicates, 4249 articles remained for title and abstract screening by the reviewing co-authors (TO, FB, and KF).

**Figure 1:**
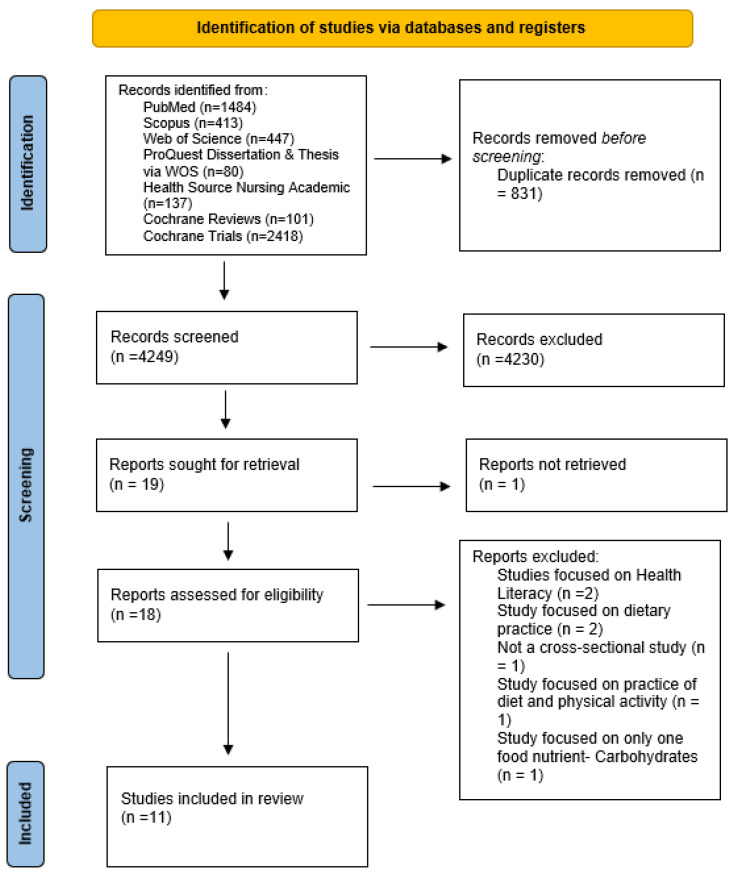
Study selection flowchart based on PRISMA guidelines.

Following this screening process, 4231 articles were excluded due to not meeting the eligibility criteria, which primarily include exploratory studies assessing the NK and/or NL of pregnant women; the blind agreement rate was over 96% among the three reviewers. As a result, 18 articles advanced to the full-text screening stage. Of these, 11 met all the eligibility requirements for the study based on the study’s inclusion and exclusion criteria. All 11 articles were included in the final analysis; Figure 1 depicts the flowcharts of how the studies were selected.

### 2.4. Data Extraction and Summarizing

The information extracted from articles that met the eligibility criteria included the title, DOI number, research focus, main findings, methodology, sample size, key measure (if applicable), study location, year, and personal reflections. These data were compiled into an Excel spreadsheet and reviewed by the study team for approval. Based on the analysis, we identified a total of nine relevant themes related to factors impacting the NK and NL of pregnant women across the various studies; only one study assessed NL in the United States.

## 3. Results

### 3.1. Characteristics of Included Studies 

In the full-text screening phase of the review, 18 articles were assessed, of which, 11 met the inclusion criteria (see Table 1). Two of these studies were conducted in Ethiopia [14,15], and there was one article each from Ghana [16], Pakistan [17,18], Egypt [18], Ireland [19], India [20], the United States (US) [2], Kenya [21], Tanzania [22], and Turkey [23]. NK or NL was treated as an outcome variable in nine of the studies [2,14,15,16,17,18,19,21,23] and as an independent variable in the remaining studies [20,22]. Only one [23] of the studies directly tested the association between NK/NL and adverse maternal and neonatal outcomes.

All the included studies utilized survey-based methodologies. Three employed previously validated instruments [2,16,23], while six developed their own survey instruments [15,17,18,19,20,22] The validated instruments used were the Nutrition Literacy Assessment Instrument (NLit) [24] and the General Nutrition Knowledge Questionnaire (GNKQ) [25]. Among the self-developed instruments, one adapted a tool from a prior study and applied a “translation and back-translation” process, followed by pilot testing and reliability assessment using Cronbach’s alpha. This was performed to ensure linguistic and cultural appropriateness for the Kaffa-speaking pregnant population in the Kaffa Zone of Southern Ethiopia [15]. Another study used self-developed items without citing a source but conducted a pretest for validation. One study validated its tool through expert review, while another relied solely on self-testing. One study employed a comprehensive approach, including expert review, pilot testing, and reliability testing with Cronbach’s alpha. Another combined a pretest with translation and back-translation. For the remaining two studies, no details were provided regarding the source or validation of their survey instruments [14,22].

All the studies were cross-sectional in design. However, only one focused on NL [2], while the remaining ten explored NK. Across the different studies, there was no uniformity in the measurement and assessment of NK, which was attributed to the use of different tools and the lack of a global standardized survey to assess NK or NL specifically for pregnant women. The study populations varied across the included studies, with sample sizes of 112 [2], 130 [16], 322 [14], 334 [19]; two studies had a sample size of 338 [22,23]; and the others had sample sizes of 372 [17], 378 [15], 446 [20], 468 [18], and 979 [21].

**Figure 2:**
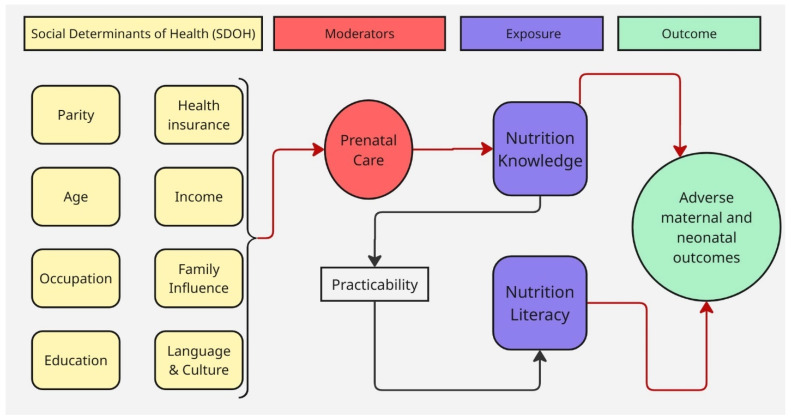
Key factors influencing nutrition knowledge (NK), and nutrition literacy (NL) based on reviewed literature. Prenatal care appointments are the primary source of NK, while practicability access are major determinants of NL. Adverse maternal and neonatal outcomes are highlighted as potential consequences of inadequate NK and NL.

This review highlights the various factors influencing NL and NK among pregnant women. Figure 2 provides a visual representation of these factors, along with the key determinants that amplify or mitigate their impact on NL and NK levels. Table 2 provides definitions of key terminology related to the concepts of NK and NL and their assessment tools.

### 3.2. Findings from the Reading

Figure 2 depicts the contributing factors for NK and NL among pregnant women that were identified in all the included studies. The findings from this review indicate that the level of NK and NL is generally low among pregnant women as this was the case across all 11 studies [2,18,22]. Similarly, social determinants of health (SDOH) and demographic factors emerged as major contributors to low NK and NL levels among pregnant women. Among the 11 studies reviewed, 8 reported a correlation between education and NK [14,15,17,18,19,20,21,23], and 5 studies found associations between income and both NK and NL [2,14,17,18,23]. Three studies identified links between occupation and NK [14,15,23], while four studies reported associations between age and NK [18,20,25,26]. One study found a correlation between health insurance and NL [2], and four studies reported associations between family influence and NK [17,18,21,23]. Additionally, two studies found a correlation between language and culture and NK and NL [2,18], and five studies reported a relationship between parity and NK [15,17,19,22,23]. These findings highlight the multifaceted influence of SDOH factors on nutritional understanding among pregnant women.

#### 3.2.1. Education

In one of the included studies, there was a positive correlation between educational attainment and levels of NK, with a median knowledge level of 80% even when accounting for other factors such as income, attitude, and parity [19]. In another study, pregnant women with higher education were 4.5 times more likely to have elevated NK levels compared to those with lower education. This increased knowledge was linked to greater access to credible information sources on optimal nutrition during pregnancy [23].

In addition to this, several studies emphasized the influence of education of the pregnant woman’s partner on NK. Two of the three studies affirmed that having an educated partner was linked to enhanced NK for pregnant women and this was found to be statistically significant [17,20,23]. However, a pregnant woman’s own educational level was found to have a more significant impact [20,23]. In addition to this, educational disparities were found to be associated with the residential location of the pregnant woman, which influenced her access to resources and prenatal care, and ultimately negatively impacted her level of NK [16,27]. In contrast to these, one study that primarily consisted of participants with lower educational attainment found no statistically significant relationship between education level and NL [2]. An Egyptian study found that higher educational levels were associated with a reduced prevalence of belief in nutritional myths [16]. Two additional studies established that education influenced the ability to apply NK in practice [9,28].

#### 3.2.2. Income

Income was also identified to have an impact on NK and NL among pregnant women with multiple studies consistently showing that pregnant women with higher earnings have higher NK and NL and this was found to be statistically significant [2,14,17,18,23]. One study found that individuals with higher income were 5.953 times more likely to have good NK compared to those with a lower income [14]. In another study, 46.2% of low-income pregnant women were found to have poor NK, while none of the high-income study participants reported poor NK [17]. Additionally, a US-based study showed that low-income individuals were 2.74 times more likely to have low NL compared to those with a higher income [2]. Also, a modest increase in income was associated with improved NK, emphasizing the potential benefits and importance of economic interventions for maternal health [14]. Economic stability was also found to enhance a pregnant woman’s ability to adopt beneficial nutritional practices and thus influence their NK and NL [14,15,18].

Four studies included a high-income cutoff, with the thresholds differing between studies. These cutoffs were based on country-specific income ranges rather than a standardized measure [2]. Across these studies, a higher income was shown to enable pregnant women to access diverse food options, educational resources, and health services, further facilitating NK comprehension and NL application [16]. Increased income was also associated with greater dietary diversity due to higher purchasing power [16]. A low annual household income was also associated with lower NL, as financial strain restricted access to essential resources [2]. Programs like Women, Infants, and Children (WIC) aim to address these income-related barriers in the US [2].

#### 3.2.3. Occupation

Similar to income, occupation, which includes employment status and job type, also impacts NK and NL levels among pregnant women. Across the three studies, professional workers consistently showed higher NK compared to unemployed women [15,23]. For instance, merchants and government employees were 7.02 and 6.05 times more likely, respectively, to have better NK than homemakers [15]. Another study reported significantly higher NK scores among professional workers (*p* = 0.018) [23], while a third study found no statistically significant association after adjusting for other factors [14]. In the case of job type, a study conducted in Ethiopia showed that pregnant women whose husbands were employed displayed better NK than those whose husbands were unemployed [14]. In the case of the job type of a pregnant woman, a few studies found that those with formal employment, white-collar positions, and positions in private enterprises displayed higher NK when compared to homemakers or those in non-formal jobs [14,15,23].

Also, pregnant women engaged in multiple jobs or agricultural work, which are known to be time-demanding and physically exhausting, often face obstacles in acquiring NK due to time and resource constraints [22]. The combined burden of meeting increased economic demands while managing their pregnancy further limits the time available for seeking preventive healthcare and attending prenatal care, which is a major source of NK through the information provided by healthcare professionals [22]. Two studies highlighted that occupation independently influenced NK, even when controlling for education and income [14,15].

#### 3.2.4. Age

Age was identified by the included studies as a factor associated with NK among pregnant women, with statistically significant findings across all studies [17,18,21,23]. One study reported that 69% of women aged 31–37 had good NK compared to 32% of those aged 17–23 [17]. Another found that NK scores increased by 0.04 points for each additional year of age, highlighting a consistent trend of improved NK with increasing age [21]. The studies conducted in Pakistan, Egypt, Kenya, and Turkey revealed that older pregnant women demonstrated higher NK compared to their younger counterparts [17,21,23]. Only the study conducted in Pakistan specified an age cutoff for older women, defining it as 31 years or older [17]. This disparity could be attributed to the accumulation of life experiences, exposure to health information, and previous pregnancy experience.

#### 3.2.5. Health Insurance

The impact of health insurance on NL was sparingly reported across the included studies. However, the study in the US found that inadequate health insurance coverage was linked to lower NL among pregnant Latina women, with limited access to essential NL resources associated with poor prenatal care; uninsured pregnant women were found to be 7.37 times more likely to have low NL compared to those who are insured [2]. Additionally, low-income pregnant women often faced challenges in obtaining health insurance, a situation described as “financial toxicity,” which further hindered access to preventative services and negatively impacted NL and pregnancy outcomes [2].

#### 3.2.6. Language and Culture

Language and culture were also identified as important factors influencing the NK and NL of pregnant women [2,18] and a study found that pregnant women who preferred Spanish as their primary language were 3.03 times more likely to have low NL [2]. For example, a study conducted in Egypt found that culturally specific beliefs significantly shaped the NK of pregnant women. In that study, more than half of the participants believed at least one myth related to nutrition during pregnancy. Additionally, 60% of the participants reported that their primary sources of NK were friends and family [18]. These strong cultural influences can undermine the effectiveness of nutrition counseling provided during prenatal care as healthcare professionals often face challenges when addressing deeply rooted beliefs [29]. Similarly, language also plays a significant role in NL, as demonstrated by a study in the US which found that Spanish-speaking pregnant women had lower levels of NL. Their cultural background was also found to influence their NL [2]. It is predicted that language and cultural barriers between Spanish-speaking pregnant women and their English-speaking healthcare providers may their limit access to and understanding of the nutritional education they receive. This information is often primarily available in English and lacks cultural accommodation [2,30,31,32].

#### 3.2.7. Family Influence

A few studies in this review found that family members had a significant influence on the NK and NL of pregnant women [17,18,21,23]. Families were identified as a primary source of NK for pregnant women, promoting intergenerational knowledge transfer [17] and 60% of the study participants in one of the studies relied on their family and friends for nutritional advice [18]. Families can either reinforce or propagate misconceptions about nutrition. One study revealed that pregnant women in nuclear families had higher NK scores than those in extended families [23], suggesting that family structure might influence dietary knowledge. Beyond spouses, older people also influence pregnant women’s dietary habits, with family and friends serving as major NK sources for them [18], although not always in alignment with evidence-based guidelines. In a study in Kenya, family composition factors such as marital status, household income, education level, and family size were found to influence pregnant women’s NK [21]. These findings emphasize the importance of family-centered approaches to enhance NK and NL among pregnant women [17,18,21,23].

#### 3.2.8. Parity

In this review, parity emerged as a key factor associated with NK, with multiparous women consistently demonstrating higher NK levels compared to primigravida women (those experiencing their first pregnancy) [15,17,19,22,23]. Two studies reported a statistically significant association between higher NK and multiparity [17,23]. The lower NK observed among primigravida women was attributed to their limited prior exposure to pregnancy-related health information [15,19,23]. Meanwhile, multiparous women benefited from prior interactions with healthcare providers, repeated prenatal visits, and experiential learning during previous pregnancies, all of which contributed to their enhanced NK [15,17,19,22,23]. In contrast, a study in Tanzania found that primigravida women demonstrated higher NK, potentially due to increased attention and advice from healthcare providers and social networks, as well as a stronger motivation to adhere to nutritional guidance [22]. Another study, however, recommended providing targeted nutritional counseling for primigravida women, who may be in greater need of NK education [19].

#### 3.2.9. Pregnancy Complications

One study found out that pregnant women at risk for preterm labor, premature rupture of membranes, preeclampsia, gestational hypertension, or eclampsia, as well as those receiving ongoing treatment, were found to have poorer NK compared to those with healthy pregnancies [23]. Although this study did not establish causality, the evidence suggests that poor NK may be associated with these pregnancy complications. Based on this finding, the study further recommends prioritizing nutrition counseling, the professional guidance provided for pregnant women through prenatal care appointments during the pre-pregnancy stage and early in the first trimester [23].

## 4. Discussion

In this scoping review, we identified 11 studies that assessed NK or NL. Of the included studies, nine that assessed NK determined that NK was low among pregnant women [14,15,17,18,19,20,21,22,23] while one study found pregnant women’s NK to be moderate [28]. Similarly, the singular paper that assessed NL found it to be low among pregnant women [2]. Table 1 presents a comprehensive summary of each of the included studies while Table 2, provides terminology definitions. 

In the studies we reviewed, the nine factors that were found to influence the NK and NL of pregnant women were education [2,14,15,16,17,18,20,21,22,23], income [2,14,17,18,21,23] occupation [14,15,21,23], age [17,18,20,21,23], health insurance [2], language and culture [2], family situation [18,23], parity [14,17,19,23], and prior pregnancy complications [14]. Prenatal care was also identified as an important factor that positively influences NK among pregnant women [15,16,17,18,22,23]. This association is largely attributed to the nutrition counseling typically provided during prenatal visits, which enhances pregnant women’s NK [16,17,18,22].

This scoping review found that there is a paucity of studies examining other confounding factors such as health insurance, language and culture, family influence, and history of pregnancy complications. To address this gap, future studies should consider a broader range of confounding variables to better understand their impact on NK and NL among pregnant women. Furthermore, the limited assessment of NK and NL globally is concerning, especially given the high prevalence of nutrition-related pregnancy complications [33,34]. This review recommends integrating services that assess NK and NL to determine whether these issues represent a broader maternal and child health crisis.

Food access plays a critical role in shaping NK and, more significantly, NL [35]. Availability of diverse food options, especially in food deserts and other underserved areas, has been identified as a major health disparity [36,37]. These challenges are most pronounced among low-resource geographic locations [27,28] and hence, grossly affect the pregnant women in these locations. This study recommends that future research independently examine the impact of food access and food insecurity on NK and NL. Additionally, studies should explore how food access and insecurity act as modifiers influencing the prevalence of low NK and NL.

We only found one study assessing the NL of pregnant women [2]. NK is typically measured using the General Nutrition Knowledge Questionnaire (GNKQ), a validated survey that evaluates knowledge of dietary recommendations, food group classifications, healthy food choices, and the relationship between diet and disease [25]. In contrast, NL is assessed using the Nutrition Literacy Assessment Instrument (NLit), a validated tool that goes beyond knowledge and comprehension to evaluate the ability to apply nutrition information in real-world contexts, such as interpreting food labels and making informed food choices [24]. The conceptual distinction between NK and NL underscores the importance of assessing NL independently. One of the included studies provided further evidence that NL extends beyond NK by encompassing essential skills that enable pregnant women to navigate food options effectively, supporting both their own and the fetus’ health and development [2]. This study recommends prioritizing the assessment of NL among pregnant women.

Although originally developed for the general population, these validated tools have demonstrated applicability in pregnant populations [2,23]. However, to more effectively address the distinct nutritional demands of pregnancy, it may be necessary to adapt and refine these instruments by incorporating pregnancy-specific content. Furthermore, the development of innovative intervention programs targeting NK and NL should prioritize the use of revalidated or newly tailored tools. These tools will be essential for accurately evaluating the efficacy and impact of NL-focused interventions specifically designed for pregnant women.

This review highlights a notable paucity of research focused specifically on the assessment of NL among pregnant women. One contributing factor is the frequent conflation of NL with general health literacy in research [11]. While health literacy is important during pregnancy, NL holds a distinct role as it enhances self-efficacy, adherence to nutritional advice, and the prevention and management of pregnancy complications [11]. Moreover, NL is essential for enabling pregnant women to make informed dietary decisions that positively impact both maternal and fetal outcomes [26,38]. Based on this, the assessment of NL is crucial in reducing the prevalence of adverse maternal and neonatal outcomes.

Another gap in the data that was uncovered by this review was the association between NL and NK and pregnancy outcomes. We only found one study that reported lower NK among pregnant women with complications (preterm labor and HDP) [23], and we did not find any studies evaluating the association between GDM and NK or NL. The prevalence of common obstetric complications such as GDM and HDP has significantly increased in the US in recent years despite advancements in healthcare support [7,39]. Studies have also shown that these complications are linked to increased maternal and infant morbidity and mortality rates [15,20,23,40,41]. Beyond the direct health consequences, these metabolic–cardiovascular conditions impose a significant emotional burden on families and place substantial financial strain on both households and the healthcare system [42]. For instance, one study reported an excess cost of US Dollar (USD) 2.18 billion beyond standard maternal and infant healthcare expenses [42]. Since nutrition is a major risk factor in metabolic and cardiovascular health, assessing the NL of pregnant women is essential. Understanding the connection between NL and these complications could support a shift toward preventive care, particularly through the provision of universal nutritional counseling. According to a Center for Disease Control and Prevention (CDC) report, preventive care has long been recognized as a cost-saving strategy, with nutrition identified as a key component in disease prevention [43]. This aligns with the broader priorities of public health principle of the “prevention paradox”, which suggests that a preventive measure may offer substantial benefits to the overall population while providing only modest advantages to vulnerable or targeted populations, which in this case is pregnant women [44]. Therefore, improving NL may seem incremental for any single pregnant woman, but the collective impact on reducing adverse maternal and neonatal outcomes, healthcare costs, and long-term maternal and infant health outcomes is significant.

This review underscores the urgent need for US-based research examining NL among pregnant women and its association with adverse maternal and neonatal outcomes. Addressing this gap could generate critical evidence to support NL-focused interventions, inform public health strategies, and guide policy development. Integrating NL into maternal health efforts could enhance nutrition behaviors, improve pregnancy outcomes, and promote a more preventive, cost-effective healthcare model.

## 5. Conclusions

### Study Strength and Limitations

One of the strengths of the studies included in this scoping review is the use of validated instruments in three of the included studies [2,16,23]. One study used the validated NLit survey instrument which was used to measure the NL of pregnant women [2,24]. The two other studies used the G-NKQ validated survey instrument to assess the NK of pregnant women [16,23,45]. From a global perspective, this study provides a clear distinction between NK and NL, highlighting how they complement each other in practice. It also clarifies how health literacy differs from both concepts, raising awareness of the unique roles that NK and NL play in influencing pregnancy outcomes. Additionally, the findings offer a better understanding of the role of moderators, particularly prenatal care, in enhancing NK and NL among pregnant women. One limitation of this study is the out of the 11 included studies, only 1 study was based in the US. This limitation calls for more studies based in the US and other high-income countries. Also, and generally, there is a limited number of studies assessing NL among pregnant women, both in the US and globally. Additionally, few studies have examined the impact of NK on pregnancy outcomes, with none in the US. This highlights the need for more studies to assess NL among pregnant women and explore its relationship with pregnancy complications. Such research is essential to inform evidence-based practices and develop targeted care strategies for pregnant women, ultimately aiming to reduce the prevalence of nutrition-related pregnancy complications.

## Figures and Tables

**Table 1:** A comprehensive summary of the included studies.

Study Title and Year	Years Study Was Conducted	Study Aim	Study Design and Methodology	Sample Size	Study’s Primary Focus	Country	Study’s Major Findings
Pregnant mothers’ knowledge, attitude, practice and its predictors towards nutrition in public hospitals of Southern Ethiopia: A multicenter cross-sectional study [15]	2021–2022	To assess pregnant women’s nutrition knowledge, attitudes, and practices, as well as the factors that influence them	Cross-Sectional Survey	378	Nutrition Knowledge	Ethiopia	Pregnant women’s nutritional knowledge (39.1% have low levels), attitude (40.5% have unfavorable attitudes), and practice (47.7% have poor practices) are low and are significantly associated with their educational status; various sociodemographic factors like occupation, parity, and income, as well as attitude itself, influence these outcomes, suggesting a need for enhanced nutritional counseling.
Nutritional knowledge, attitude and practices among pregnant women who attend antenatal care at public hospitals of Addis Ababa, Ethiopia [14]	2015–2018	To assess the nutritional knowledge, attitudes, and practices of pregnant women who attend antenatal care at public hospitals	Cross-Sectional Survey	322	Nutrition knowledge	Ethiopia	The study revealed low nutritional knowledge (27%), poor attitudes (48.4%), and poor practices (34.5%) among pregnant women. Educational status, family income, and attitude were significantly associated with nutritional knowledge.
Income Level but Not Nutrition Knowledge Is Associated with Dietary Diversity of Rural Pregnant Women from Northern Ghana [16]	2020–2021	To evaluate the nutrition knowledge, attitudes, and dietary diversity of pregnant women and to investigate the sociodemographic factors that determine their dietary diversity	Cross-Sectional Survey	130	Nutrition knowledge	Ghana	The study found that pregnant women’s nutrition knowledge was limited (mean score: 2.65 out of 5) but it is not a significant determinant of dietary diversity.
Impact of Education on Knowledge of Women Regarding Food Intake During Pregnancy: A Hospital Based Study [17]	2016–2020	To evaluate the nutritional knowledge of pregnant women concerning their food intake during pregnancy and to determine if there is an association between their level of education and their nutritional knowledge	Cross-Sectional Survey	378	Nutrition Knowledge	Pakistan	The study’s major findings indicate that the nutritional knowledge of pregnant women is generally limited. NK was found to be associated with education level and socioeconomic status.
Nutrition and diet myths, knowledge and practice during pregnancy and lactation among a sample of Egyptian pregnant women: a cross-sectional study [18]	2022–2024	To assess the nutritional knowledge, belief in nutritional myths, and practices of pregnant women	Cross-Sectional Survey	468	Nutrition Knowledge	Egypt	This study found that the nutrition knowledge among older pregnant women was higher than that of younger pregnant women.
Access to nutrition advice and knowledge, attitudes and practices of pregnant women in Ireland: A cross—sectional study [19]	2024	To explore the relationship between pregnant women’s access to nutrition advice, their nutrition knowledge, and their attitudes and practices regarding nutrition	Cross-Sectional Survey	446	Nutrition Knowledge	Ireland	This study found that pregnant women with previous nutrition counseling had significantly better NK scores than those without (80.0% vs. 73.3%).
Oral health, nutritional knowledge, and practices among pregnant women and their awareness relating to adverse pregnancy outcomes [20]	2015–2016	To assess the nutritional knowledge of pregnant women and to evaluate their oral health-related awareness and practices	Cross-Sectional Survey	112	Nutrition Knowledge	India	The study’s findings indicate that pregnant women had limited specific nutritional knowledge. A minority of participants correctly knew the meaning of food (40.1%), the importance of food during pregnancy (45.5%), what a balanced diet is (47%), and the difference between healthy and unhealthy foods (43.9%).
Nutrition Literacy Among Latina/x People During Pregnancy Is Associated With Socioeconomic Position [2]	2018–2022	To assess the nutrition literacy level of Latina/x people during pregnancy and to explore the association between nutrition literacy and socioeconomic position (SEP)	Cross-Sectional Survey	979	Nutrition Literacy	United States	The study found that a majority of the 112 participating pregnant Latina/x people had a low nutrition literacy level, with a mean score of 24.7 (a score ≤ 28 indicates low nutrition literacy).
Health and nutrition knowledge, attitudes and practices of pregnant women attending and not-attending ANC clinics in Western Kenya: a cross-sectional analysis [21]	2011–2013	To compare the nutrition knowledge and health knowledge, attitudes, and practices (KAP) of pregnant women who attended antenatal care clinics versus those who did not	Cross-Sectional Survey	338	Nutrition Knowledge	Kenya	The study found no significant difference in NK between pregnant women who attended antenatal care clinics and those who did not, with a mean Nutrition Knowledge Score (NKS) of 4.6 out of 11 for both groups.
Dietary diversity and associated factors among women attending antenatal clinics in the coast region of Tanzania [22]	2020–2024	To assess dietary diversity and its associated factors, which explicitly included nutrition knowledge, among pregnant women attending antenatal care	Cross-Sectional Survey	369	Nutrition Knowledge	Tanzania	The study found that the overall level of nutrition knowledge among pregnant women was low, with only 18% (number = 59) considered to have a high level of nutrition knowledge.
Nutrition perspective from the view of pregnant women: their understanding of fetal well-being relative to their diet [23]	2019	To assess the nutritional habits and the levels of nutritional knowledge among pregnant women	Cross-Sectional Survey	338	Nutrition Knowledge	Turkey	The study found that NK was significantly lower in women with pregnancy complications like preeclampsia (total score of 51.89) and higher in those with more education (total score of 63.04 for those with undergraduate/graduate degrees).

**Table 2:** Definitions of concepts related to nutritional knowledge and literacy and their assessment scales.

Concept	Definition
Nutrition Knowledge	NK refers to the factual understanding a pregnant woman has about nutrition, including awareness of dietary guidelines, sources of nutrients, everyday food choices, and the relationship between diet and disease. It also includes the ability to recognize, recall, and apply food- and nutrition-related terminology [8,9]. The GNKQ is an instrument used to assess an individual’s level of nutrition knowledge [25].

Nutrition Literacy	NL is the ability to interpret and use NK to make informed food choices and maintain a healthy diet. It includes practical skills and decision-making beyond basic knowledge [2,9,10]. It is assessed with the NLit instrument used to assign a score for an individual’s nutrition literacy level [24]
Health Literacy	Health literacy is the ability of individuals to obtain, process, and understand basic health information and services needed to make informed decisions about their overall well-being. It encompasses a broad range of topics beyond nutrition, including physical activity, disease prevention, medication management, and navigating healthcare systems [11].

## Data Availability

The original contributions presented in this study are included in the article. Further inquiries can be directed to the corresponding author.

## Abbreviations

The following abbreviations are used in this manuscript:

GDM	Gestational Diabetics Mellitus
GNKQ	General Nutrition Knowledge Questionnaire
HDP	Hypertensive Disorders of Pregnancy
NK	Nutrition Knowledge
NL	Nutrition Literacy
OSF	Open Science Framework
US	United States
WIC	Women, Infants, and Children

## Appendix A

The following search terms were used:

#1. ((premature birth [mesh] OR “preterm birth*” [tiab] OR “early birth” [tiab] OR prematurity [tiab] OR “preterm delivery” [tiab] OR “preterm labor” [tiab]) OR (pregnancy complications [mesh] OR (hypertension [mesh] AND pregnancy [mesh])OR “preeclampsia” [tiab] OR “pre-eclampsia” [tiab] OR eclampsia [tiab] OR “hellp syndrome” [tiab] OR “gestational hypertension” [tiab] OR “hypertensive disorders of pregnancy” [tiab] OR “gestational diabetes” [tiab])) AND ((“nutritional literacy” [tiab:~2] OR “food literacy” [tiab:~2] OR “nutrition literacy” [tiab:~2] OR “food agency” [tiab] OR “nutri* education” [tiab] OR “diet* education” [tiab] OR “nutrition* awareness” [tiab] OR dietetics [mesh] OR ((“health literacy” [mesh] OR literacy [mesh]) AND (nutrition [mesh] OR nutrition [tiab] OR “feeding behavior” [mesh] OR food [mesh] OR food [tiab] OR diet [mesh] OR “eating pattern*” [tiab] OR diet [tiab] OR nutrients [mesh] OR nutrients [tiab]))
#2. ((prenatal care [mesh] OR “prenatal care” [tiab] OR “maternal health” [tiab] OR “perinatal care” [tiab] OR “pregnancy care” [tiab] OR “prenatal period” [tiab] OR “perinatal period” [tiab] OR “gestation* period” [tiab])) AND (((“nutritional literacy” [tiab:~2] OR “food literacy” [tiab:~2] OR “nutrition literacy” [tiab:~2] OR “food agency” [tiab] OR “nutri* education” [tiab] OR “diet* education” [tiab] OR “nutrition* awareness” [tiab] OR dietetics [mesh] OR ((“health literacy” [mesh] OR literacy [mesh]) AND (nutrition [mesh] OR nutrition [tiab] OR “feeding”
#3. (“Health Knowledge, Attitudes, Practice” [Mesh] OR “Health education” [Mesh] OR “health literacy” [tiab]) AND (nutrition [mesh] OR “Prenatal Nutritional Physiological Phenomena” [Mesh] OR nutritional status [mesh] nutrition [tiab] OR “feeding behavior” [mesh] OR diet [mesh] OR “eating pattern*” [tiab] OR diet [tiab] OR “diet* pattern*” [tiab] OR nutrients [mesh] OR nutrients [tiab] OR food [mesh] OR food* [tiab])) OR (“nutri* literacy” [tiab] OR “nutri* education” [tiab] OR “nutri* knowledge” [tiab] OR “nutri* counsel*” [tiab] OR “nutrition education and counselling” [tiab] OR “food literacy” [tiab] OR “food knowledge” [tiab] OR “food education” [tiab] OR “diet* knowledge” [tiab] OR “diet* education” [tiab] OR “food education” [tiab])) AND (“pregnancy complications” [MeSH Terms] OR “pregnan* complicat*” [Title/Abstract] OR “abnorm* pregnan*” [tiab] OR “pregnan* abnorm*” [tiab] OR “fetal abnorm*” [tiab] OR “neonatal abnorm*” [tiab] OR “birth defect*” [tiab] OR “gestational diabetes” [Title/Abstract] OR “eclampsia” [Title/Abstract] OR “pre-eclampsia” [Title/Abstract] OR “preterm birth” [Title/Abstract] OR “premature birth” [Title/Abstract] OR “fetal growth restriction” [Title/Abstract] OR “low birth weight” [Title/Abstract] OR “hellp syndrome” [Title/Abstract] OR “hemolysis, elevated liver enzymes, and low platelet count” [tiab] OR “stillbirth” [Title/Abstract] OR “abortion” [Title/Abstract] OR “reproductive complication*” [tiab] OR “obstet* complicat*” [Title/Abstract] OR “birth complicat*” [Title/Abstract] OR “perinatal complicat*” [Title/Abstract] OR “labor complicat*” [Title/Abstract] OR “pregnancy induced hypertension” [Title/Abstract] OR “hypertensive disorders of pregnancy” [Title/Abstract] OR “fetal growth retardation” [Title/Abstract] OR “macrosomia” [Title/Abstract] OR “miscarriage*” [Title/Abstract] OR “pregnancy loss*” [tiab] OR “maternal mortality” [Title/Abstract] OR “fetal mortality” [Title/Abstract] OR “neonatal mortality” [Title/Abstract])
  behavior” [mesh] OR food [mesh] OR food [tiab] OR diet [mesh] OR “eating pattern*” [tiab] OR diet [tiab] OR nutrients [mesh] OR nutrients [tiab])))
